# Involvement of Mrs3/4 in Mitochondrial Iron Transport and Metabolism in *Cryptococcus neoformans*

**DOI:** 10.4014/jmb.2004.04041

**Published:** 2020-05-21

**Authors:** Yoojeong Choi, Eunsoo Do, Guanggan Hu, Mélissa Caza, Linda C. Horianopoulos, James W. Kronstad, Won Hee Jung

**Affiliations:** 1 Department of Systems Biotechnology, Chung-Ang University, Anseong 17546, Republic of Korea; 2 The Michael Smith Laboratories, Department of Microbiology and Immunology, University of British Columbia, Vancouver BC, V6T 1Z4, Canada

**Keywords:** *Cryptococcus neoformans*, iron, iron transport, mitochondria, Mrs3/4

## Abstract

Mitochondria play a vital role in iron uptake and metabolism in pathogenic fungi, and also influence virulence and drug tolerance. However, the regulation of iron transport within the mitochondria of *Cryptococcus neoformans*, a causative agent of fungal meningoencephalitis in immunocompromised individuals, remains largely uncharacterized. In this study, we identified and functionally characterized Mrs3/4, a homolog of the *Saccharomyces cerevisiae* mitochondrial iron transporter, in *C. neoformans* var. *grubii*. A strain expressing an Mrs3/4-GFP fusion protein was generated, and the mitochondrial localization of the fusion protein was confirmed. Moreover, a mutant lacking the *MRS3/4* gene was constructed; this mutant displayed significantly reduced mitochondrial iron and cellular heme accumulation. In addition, impaired mitochondrial iron-sulfur cluster metabolism and altered expression of genes required for iron uptake at the plasma membrane were observed in the *mrs3/4* mutant, suggesting that Mrs3/4 is involved in iron import and metabolism in the mitochondria of *C. neoformans*. Using a murine model of cryptococcosis, we demonstrated that an *mrs3/4* mutant is defective in survival and virulence. Taken together, our study suggests that Mrs3/4 is responsible for iron import in mitochondria and reveals a link between mitochondrial iron metabolism and the virulence of *C. neoformans.*

## Introduction

*Cryptococcus neoformans* is a pathogenic fungus that infects humans to cause meningoencephalitis, a disease that is especially life-threatening for immunocompromised individuals. Isolates of *C. neoformans* are divided into two varieties (var.): *C. neoformans* var. *grubii* (capsule serotype A) and *C. neoformans* var. *neoformans* (capsule serotype D). The former is more frequently isolated from patients with cryptococcosis, shows higher virulence in experimental animal models, and includes the most commonly employed laboratory strain for studying virulence [1].

Iron is an essential nutrient for most organisms because it is a cofactor of many enzymes involved in numerous cellular processes. In *C. neoformans*, the importance of iron for several processes, including normal growth and pathogenesis, is well appreciated. For instance, the virulence traits of melanin synthesis and polysaccharide capsule formation are regulated by environmental iron [2]. Therefore, maintaining proper intracellular iron levels is essential for *C. neoformans* to meet physiological needs, particularly during host colonization. However, the level of available iron in the mammalian host is extremely low, since the majority of iron is bound to iron-binding proteins such as transferrin, lactoferrin, and hemoglobin. Upon infection, *C. neoformans* must overcome host iron sequestering systems to acquire iron for successful proliferation. To this end, the fungus has developed elaborate iron uptake and utilization systems, including high-affinity reductive iron uptake, siderophore transport, and heme utilization systems [3-6].

Iron acquired from the extracellular environment must be tightly regulated to avoid toxicity and it must be processed for cellular utilization by sophisticated mechanisms. In eukaryotic cells, mitochondria are not only a major consumer of iron but also play global iron regulatory roles in numerous iron metabolic processes and homeostasis [7-10]. One of the important iron metabolic processes in mitochondria is the transformation of iron to its bioactive forms, such as an iron–sulfur (Fe-S) cluster, and the mitochondria are the sole site for this process [11-13]. A model for Fe-S cluster biosynthesis has been best described in the model yeast *Saccharomyces cerevisiae* [8, 11, 14, 15]. The mitochondrial carrier proteins, Mrs3 and Mrs4, along with the frataxin protein Yfh1, transport iron into mitochondria [16, 17], and Fe-S clusters are assembled by a scaffold protein complex in the organelle. An unidentified form of the Fe-S cluster is then exported to the cytosol by the mitochondrial export protein Atm1 and a maturation process ensues for cytosolic utilization [18, 19].

We previously identified and characterized the mitochondrial iron exporter protein Atm1 in *C. neoformans* var. *grubii,* and we demonstrated that Atm1 is required for virulence [20]. Although a role for Atm1 iron transport has been suggested, we still do not have a comprehensive understanding of the proteins responsible for iron uptake by mitochondria in *C. neoformans*; this is especially true for the *grubii* variety.

Previously, Nyhus *et al*. (2002) identified the putative homolog of the *S. cerevisiae* mitochondrial iron importers, Mrs3 and Mrs4, in *C. neoformans*. However, their study focused on a limited number of phenotypes of deletion mutants, and evidence was not provided that the identified protein was localized in mitochondria or was involved in virulence in a vertebrate host. Moreover, the study was performed using *C. neoformans* var. *neoformans* and not the laboratory strain of the *grubii* variety that is commonly employed to study virulence. Therefore, in this study, we aimed to identify and functionally characterize the mitochondrial iron import protein (the homolog of *S. cerevisiae* Mrs3 and Mrs4) in *C. neoformans* var. *grubii*. Moreover, we investigated the contribution of the mitochondrial iron importer to virulence using a murine model of cryptococcosis.

## Materials and Methods

### Strains, Growth Conditions, and Phenotypic Analysis

*C. neoformans* var. *grubii* strain H99 (serotype A; *MATα*) was used for all experiments. Strains were cultured in yeast extract-bacto peptone medium containing 2.0% glucose (YPD) or defined yeast nitrogen base medium containing 2.0% glucose (YNB). To prepare low iron YNB medium, the broth was chelated using chelex-100 (Biorad, USA), its pH was adjusted to 6.8 by adding 3-morpholinopropanesulfonic acid (MOPS), and 100 μM of bathophenanthrolinedisulfonic acid (BPS) was added. For assessing phenotypes, cells were cultured in YPD at 30°C overnight, transferred to low iron medium, and cultured at 30°C for an additional 16 h to remove any intracellularly-stored iron. Cells were washed twice with chelated PBS, and cell numbers were determined using a hemocytometer. Serially diluted cells were spotted on plates and subsequently photographed after incubation at 30°C for 2 days.

### Mutant Construction

The *mrs3/4* knockout mutant was constructed by homologous recombination using a gene-specific disruption cassette generated by overlapping PCR. The primers used to amplify the gene specific disruption cassette, KO_1, KO_2, KO_3, KO_4, KO_5, and KO_6, were used for overlapping PCR with wild-type genomic DNA and pCH233 plasmid DNA as templates [21]. The resulting disruption cassette was introduced into the wild-type strain using biolistic transformation, as previously described [22]. Positive independent transformants were confirmed by PCR and Southern blot analysis using a probe generated with the primers Probe_F and Probe_R [23]. The *MRS3/4* coding region was amplified using the Forward_HindIII and Reverse_BamHI primers and cloned into the plasmid pWH091. The resulting plasmid was linearized by KpnI digestion and introduced into the *mrs3/*4 mutant by biolistic transformation. All primers used in this study are listed in Table S1.

### Fluorescence Microscopy

To visualize the subcellular location of the Mrs3/4-green fluorescence protein (GFP) fusion protein, the strain expressing the protein was grown in YPD medium at 30°C overnight. Cells were washed twice with iron-chelated water and resuspended with low iron YNB medium, followed by incubation at 30°C for 6 h. Mitotracker Red CMXRos (Thermo Fisher Scientific, Korea) was added to YNB medium at a final concentration of 100 nM to stain mitochondria. The cells were incubated at 30°C for 30 min and visualized using an Axioplan 2 imaging system (Zeiss, Germany) at 1,000× magnification. Differential interference contrast (DIC) and fluorescence images were obtained using the Metamorph imaging software (Universal Imaging Corporation, USA).

### Mitochondrial Isolation

Isolation of mitochondria was performed using a differential centrifugation method [24]. Briefly, the strains were grown in YPD overnight at 30°C and harvested by centrifugation at 6,000 rpm for 5 min. The resulting pellet was washed twice with distilled H_2_O, resuspended in buffer containing 100 mM Tris-SO_4_, pH 9.4 and 10 mM dithiothreitol (DTT), and incubated for 10 min at 30°C. The cells were harvested by centrifugation, washed with spheroplast buffer containing 20 mg/ml of lysing enzyme (Sigma, USA), 1 M sorbitol, and 20 mM potassium phosphate buffer pH 7.4, and incubated at 37°C for 1 h. The harvested cells were washed twice with 1.2 M sorbitol, resuspended with homogenization buffer containing 0.6 M mannitol, 10 mM Tris-Cl, pH 7.4, 0.1% BSA and 1 mM phenylmethylsulfonyl fluoride (PMSF) and lysed by vortexing for 6 min. The isolation of mitochondria from cell lysates was performed as described for *S. cerevisiae* [25].

### Determination of Intracellular Iron and Heme Levels

Mitochondrial iron levels and intracellular heme levels were respectively determined using the QuantiChrom™ Iron Assay kit (DIFE-250; BioVision, USA) and the BioVision Hemin assay kit (BioVision), following the manufacturers’ instructions.

### Aconitase Activity and TTC Overlay Assays

Zymography was performed to measure the activity of aconitase as described previously [26]. The wild-type strain and the *mrs3/4* mutant were grown in YPD overnight at 30°C, pelleted, and resuspended with triton/citrate lysis buffer (36.6 mM KCl, 22.9 mM Tris-Cl (pH 7.5), 1% Triton X-100, 1 mM of PMSF, 1 mM DTT, 2 mM sodium citrate and 0.6 mM MnCl2). The cells were lysed using a mini-bead beater, and total protein concentrations were measured by Bradford assay. Samples containing 20 μg of protein lysate were separated on a zymography gel at 160 V and 4°C for 2 h. The gel was visualized using aconitase-staining buffer and photographed as described previously [27]. The triphenyltetrazolium chloride (TTC; Sigma, USA) overlay assay to estimate activity of cell surface reductase was performed as described previously [28].

### Virulence Assay

The virulence of the wild-type strain and the *mrs3/4* mutant was assayed in a murine inhalation model of cryptococcosis using female BALB/c mice (4 to 6 weeks old) from Charles River Laboratories (Canada), as previously described [29]. Briefly, the strains were grown in 5 ml of YPD at 30°C overnight, washed twice with PBS (Invitrogen, USA), and resuspended in PBS. Groups of 10 BALB/c mice were intranasally inoculated with a suspension of 2 × 10^5^ cells in 50 μl. The health status of the mice was monitored daily post-inoculation. Mice reaching the humane endpoint were euthanized by CO_2_ anoxia. Statistical analyses of survival differences were performed using log rank tests in GraphPad Prism 5 for Windows (GraphPad software, USA). The fungal load in lungs, brain, kidney, liver, spleen, and blood in ten mice was assessed at humane endpoints of the experiment. Organs were aseptically removed, weighed, homogenized, and diluted in PBS prior to inoculation on YPD plates. Blood was retrieved from the heart and directly inoculated onto YPD plates. Plates were incubated at 30°C for 48 h and CFU counts were determined. Virulence assay protocols (protocol A17-0117) were approved by the University of British Columbia Committee on Animal Care.

## Results

### Identification of the Mitochondrial Iron Transporter Mrs3/4 in *C. neoformans* var. *grubii*

To identify potential orthologs of the *S. cerevisiae* Mrs3 and Mrs4 proteins in the *grubii* variety of *C. neoforman*s, we carried out BLASTp analysis using the fungal genome database (http://www.fungidb.org). A single gene, CNAG_02522, was identified to encode the homolog of both *S. cerevisiae* Mrs3 and Mrs4, with respective identities of 46% and 44%. CNAG_02522 was also homologous with the *Candida albicans* genes encoding the Mrs3 and Mrs4 proteins [30] and the *C. neoformans* var. *neoformans* Mrs3/4 homolog (Genbank AY048991), which was identified in a previous study [31]. Analysis of the protein sequence encoded by CNAG_02522 using Mitoprot II revealed a mitochondrial targeting sequence and a cleavage site in the N-terminal region [32]. Since no other homolog was identified, we concluded that CNAG_02522 is the only homolog of *S. cerevisiae* Mrs3 and Mrs4 and therefore designated it Mrs3/4.

### Functional Characterization of Mrs3/4

**Fig. 1:**
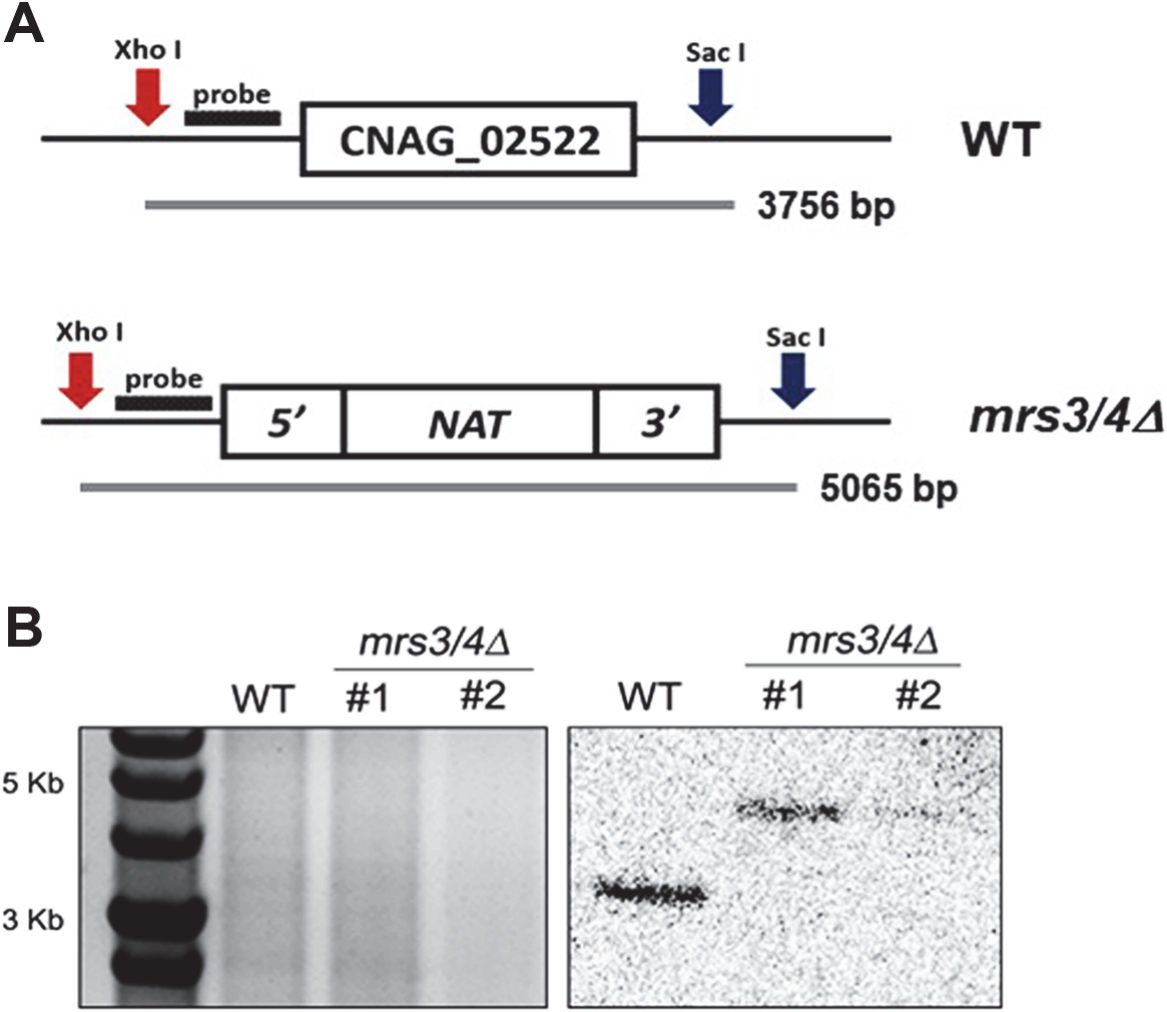
Construction of the *mrs3/4* mutant. (**A**) To confirm the disruption of *MRS3/4*, genomic DNA from the wild-type strain and the *mrs3/4* mutant was digested with Xho1/SacI and hybridized with the indicated probes. (**B**) Southern blot analysis indicated genomic deletion of *MRS3/4*. Two independent *mrs3/4* mutants (#1 and #2) were constructed, and #1 was used throughout the study.

**Fig. 2:**
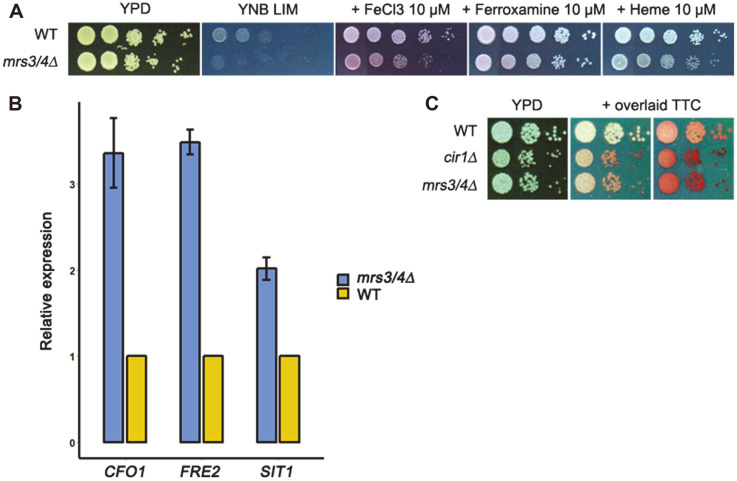
Iron transport and metabolism deficiencies in the *mrs3/4* mutant. (**A**) The growth of the *mrs3/4* mutant in low-iron YNB media (LIM) containing various iron sources was monitored. Ten-fold serial dilutions of cells (starting at 104 cells) were spotted onto the plates and incubated at 30°C for 2 days. (**B**) The transcript levels of *CFO1, FRE2*, and *SIT1* were determined using qRT-PCR. Data were normalized using *TEF2* as an internal control and represent the average from three independent experiments (with standard deviations indicated). (**C**) Measurement of ferric reductase activity was carried out using a TCC overlay assay. TCC was poured on spotted cells and plates were photographed after 10 min.

To investigate the functional characteristics of Mrs3/4 in *C. neoformans*, we constructed an *MRS3/4* knockout mutant using biolistic transformation and confirmed complete deletion of the *MRS3/4* coding region by Southern blot analysis (Fig. 1). There was no difference between the growth of the wild-type strain and the *mrs3/4* mutant in rich medium, and capsule synthesis and melanin formation in the mutant cells were also similar to the wild-type cells (data not shown). However, the *mrs3/4* mutant showed delayed growth upon iron depletion (Fig. 2A). Furthermore, the growth defect induced by iron depletion was reversed by adding exogenous iron sources, such as FeCl3, the siderophore ferroxamine, or heme, suggesting that iron transport and metabolism are deficient in the mutant (Fig. 2A). We next investigated if the deficiencies in iron transport and metabolism in the *mrs3/4* mutant are caused by altered expression of genes required for iron uptake. The *CFO1, FRE2*, and *SIT1* genes encode proteins responsible for high-affinity iron uptake or siderophore transport at the cell membrane*,* and we evaluated for their transcript levels in the wild-type strain and the mutant. The results presented in Fig. 2B show that, indeed, the *CFO1, FRE2*, and *SIT1* transcript levels were significantly increased in the *mrs3/4* mutant. We hypothesized that mitochondrial iron is depleted in the *mrs3/4* mutant cells and, consequently, the upregulation of genes involved in iron uptake at the cell surface was triggered. Upregulation of these genes was further supported by increased cell surface reductase activity as visualized with TTC overlay, a phenotype also observed in a mutant lacking the major iron regulator Cir1 that positively regulates the high affinity iron uptake system in *C. neoformans* (Fig. 2C) [33].

### Localization and Role of Mrs3/4 in *C. neoformans* var. *grubii*

**Fig. 3:**
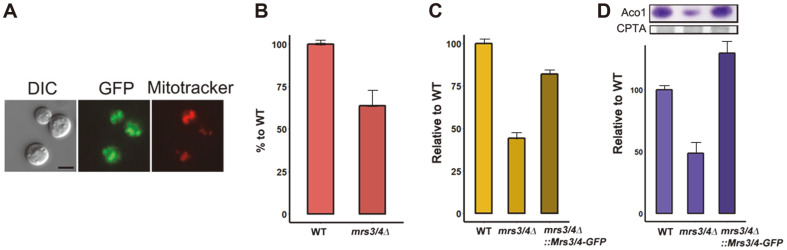
Mitochondrial localization of the Mrs3/4 protein and impaired mitochondrial iron metabolism in the *mrs3/4* mutant. (**A**)) The strain expressing the Mrs3/4-GFP fusion protein was stained with 100 nM of Mitotracker to visualize mitochondria. The scale bar represents 5 μm. Iron contents of isolated mitochondria (**B**) and total intracellular heme contents (**C**) were determined by colorimetric assays. Values indicate iron or heme contents relative to those of the wild-type and represent the average from three independent experiments, with standard deviations. (**D**) The activity of aconitase was determined using in-gel assays, and the intensity of each band was quantified. CPTA (copper phthalocyanine-3, 4', 4", 4'"- tetrasulfonic acid tetrasodium) shows equal sample loading. All experiments were carried out in triplicate.

In *S. cerevisiae*, Mrs3 and Mrs4 import iron at the inner mitochondrial membrane. To confirm mitochondrial localization of Mrs3/4 in *C. neoformans*, we constructed a strain expressing the Mrs3/4 protein fused with GFP and observed the localization of the fusion protein by fluorescence microscopy. As shown in Fig. 3A, the Mrs3/4- GFP signal overlapped with the Mitotracker (mitochondria) signal, confirming mitochondrial localization of the Mrs3/4-GFP fusion protein.

Based on the results presented above and the role of Mrs3/4 as a mitochondrial iron importer in *S. cerevisiae*, we hypothesized that the *mrs3/4* mutant might have an impaired ability to accumulate iron in mitochondria. To test our hypothesis, mitochondria were isolated from the *mrs3/4* mutant and the wild-type strain, and the mitochondrial iron content was measured and compared. Mitochondria isolated from the *mrs3/*4 mutant had lower iron levels compared with those of wild-type mitochondria (63.65% of wild-type (*p* < 0.01)) (Fig. 3B). Moreover, we measured the levels of intracellular heme, an important iron-containing molecule in mitochondria, and found that heme levels dropped to 44.24% of wild-type levels in the *mrs3/4* mutant (*p* < 0.001) (Fig. 3C). Aconitase is a mitochondrial protein that uses Fe-S clusters as a co-factor, and its enzymatic activity is a well- known marker of mitochondrial Fe-S cluster biosynthesis [20, 34]. Therefore, we measured aconitase activity in the *mrs3/4* mutant and compared it to the wild-type strain. A zymography assay revealed that the aconitase activity in the *mrs3/4* mutant was reduced to 48.76% of wild type (Fig. 3D). Expression of the Mrs3/4-GFP fusion protein in the deletion mutant complemented the defects in heme levels and aconitase activity, thereby providing support for both the impact of the *mrs3/4* deletion on the observed phenotypes and the functionality of the fusion protein. Overall, our data indicate that Mrs3/4 is a mitochondrial protein and that deletion of *MRS3/4* causes a significant deficiency in mitochondrial iron metabolism (e.g., heme and Fe-S cluster biosynthesis), which is likely due to defects in mitochondrial iron uptake.

### Analysis of the Requirement of Mrs3/4 for Virulence in a Murine Model of Cryptococcosis

**Fig. 4:**
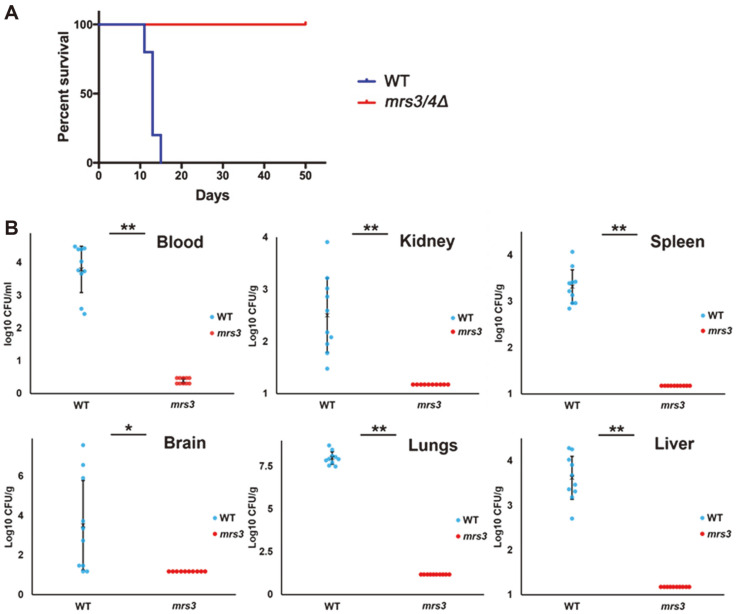
Requirement of MRS3/4 for virulence in a mouse inhalation model. (**A**) Ten female BALB/c mice were intranasally infected with each of the strains indicated, and mouse survival was monitored twice per day. The results from the assays indicate that *MRS3/4* is required for full virulence. (**B**) The distribution of fungal cells in the organs (blood, kidney, spleen, brain, lung, and liver) of infected mice. Organs from wild-type and *mrs3/4* mutant infected mice were collected at the humane endpoint of the experiment, and fungal burdens were quantified by CFUs. In all organs, differences in fungal burden between the wild-type and the *mrs3/4* mutant were statistically significant, as assessed by a nonparametric two-tailed Mann- Whitney *U* test (*p < 0.05; **p < 0.001).

Iron metabolism and mitochondrial functions are critical for the survival and virulence of *C. neoformans* within a vertebrate host [20, 35, 36]. Therefore, we examined the differences in the survival and virulence of the *mrs3/4* mutant and the wild type in a murine inhalation model of cryptococcosis. The mice infected with the *mrs3/4* mutant survived until the end of the experiments, while mice infected with the wild type survived only until 10 days post-infection (Fig. 4A). We examined reduction in the virulence of the *mrs3/4* mutant in more detail by assessing the fungal burden in the organs of infected mice. This was accomplished by measuring the number of fungal colony forming units (CFUs) present in the organs at the humane endpoint. As shown in Fig. 4B, the CFUs of the *mrs3/4* mutant were significantly reduced in each organ compared to those of the wild type, suggesting that Mrs3/4 is required for the proliferation and survival of the fungus in mice.

## Discussion

In this study, we identified and functionally characterized Mrs3/4 in the *grubii* variety of *C. neoformans.* Mrs3/ 4 is the homolog of the *S. cerevisiae* Mrs3 and Mrs4 proteins, which are solute carrier proteins located at the mitochondrial inner membrane that import iron into mitochondria [37, 38]. It has been suggested that Mrs3 and Mrs4, along with frataxin Yfh1, cooperatively provide iron for heme and Fe-S cluster biosynthesis in *S. cerevisiae* mitochondria [16, 17]. Furthermore, loss of an *Aspergillus fumigatus* homolog of *S. cerevisiae* Mrs3 and Mrs4 (MrsA) resulted in delayed growth under iron-limiting conditions and attenuated virulence [39].

Nyhus *et al*. (2002) previously identified an *MRS3/4* gene in *C. neoformans* var. *neoformans,* and found that the gene complemented mutations in the *OXY1* and *FRR1* genes, which cause constitutively high reductase activity and increased intracellular iron accumulation. The conclusion from this study was that *OXY1* and *FRR1* represent alleles of the same gene complemented by *MRS3/4*, and that the predicted amino acid sequence of *C. neoformans* var. *neoformans* Mrs3/4 was homologous to the Mrs3 and Mrs4 proteins of *S. cerevisiae*. Additionally, it was found that disruption of the *MRS3/4* gene increased reductase activity and total intracellular iron accumulation, and caused a slow growth phenotype in the mutant cells under iron-limiting conditions. While Nyhus *et al*. (2002) found increased total intracellular iron levels in the *mrs3/4* mutant, we observed significantly reduced iron levels in isolated mitochondria from the mutant. The results of these observations suggest that deletion of *MRS3/4* reduced iron accumulation in mitochondria, and that iron deprivation within mitochondria induced iron uptake at the cellular membrane leading to increased iron levels in the cytoplasm. Our results also suggested that Mrs3/4 indeed plays a role in iron transport at the interface between the cytosol and mitochondria to regulate iron compartmentalization in *C. neoformans*, and that mitochondria play a central role in controlling intracellular iron homeostasis. We should note that our findings of reduced iron levels in isolated mitochondria from the *mrs3/4* mutant, and reduced intracellular heme content and Fe-S biosynthesis in the mutant cells, agree well with the findings for the *S. cerevisiae mrs3* and *mrs4* mutants [37, 38].

Although functions related to iron transport and metabolism were observed in the study by Nyhus *et al*. (2002), experimental confirmation that Mrs3/4 is indeed a mitochondrial protein responsible for iron import was not provided. Here, we clearly showed that Mrs3/4 is localized in the mitochondria of *C. neoformans* var. *grubii* and that loss of *MRS3/4* caused growth defects in this variety under iron-limiting conditions. Deletion of *MRS3/4* also caused a significant reduction in mitochondrial iron accumulation and deficiency in mitochondrial iron metabolism (heme and Fe-S cluster biosynthesis) [11-13]. In *S. cerevisiae*, Fe-S clusters are assembled by a scaffold protein complex within mitochondria, followed by export of the molecules to the cytosol by the mitochondrial iron exporter protein Atm1, the *C. neoformans* homolog of which was identified and functionally characterized in our previous study [20]. The *C. neoformans* mutant strain lacking *ATM1* showed significantly increased mitochondrial iron accumulation, which also led to the disruption of mitochondrial iron homeostasis and impairment of the cytosolic Fe-S cluster maturation machinery. The results of the current study suggest that Mrs3/ 4, as an iron importer, may play opposing roles to Atm1 in mitochondrial iron metabolism in *C. neoformans*.

Importantly, no studies have yet investigated whether Mrs3/4 influences the survival and virulence of *C. neoformans* in a vertebrate host. Our study revealed that Mrs3/4 is required for the virulence of *C. neoformans* var. *grubii* in a murine model of cryptococcosis. Our data are in agreement with the results for *A. fumigatus* in which loss of MrsA, the homolog of Mrs3/4, resulted in delayed growth under iron-limiting conditions and attenuated virulence [39]. Taken together, the results indicate that the role of Mrs3/4 is highly conserved in pathogenic fungi and that mitochondrial iron uptake and metabolism are critical to fungal pathogenesis.

## Supplemental Materials



Supplementary data for this paper are available on-line only at http://jmb.or.kr.
