# Supplementary material for: Involvement of Mrs3/4 in Mitochondrial Iron Transport and Metabolism in Cryptococcus neoformans
Source: J Microbiol Biotechnol. 2020 May 21;30(8):1142–8. doi: 10.4014/jmb.2004.04041 (PMC7490968; doi:10.4014/jmb.2004.04041)
Supplement: Supplementary file 1 [file JMB-30-8-1142-supple.pdf]

J. Microbiol. Biotechnol.
https://doi.org/10.4014/jmb.2004.04041

Table S1. Primer used in this study

Name	Sequence (5' to 3')
KO_1	GACCGTTGTATGATTGAGCGCCG
KO_2	AATTCTGCAGATATCCATCACACTGGCGGCTGTTGAGTTGGGGGGCAATGGTG
KO_3	AATTCCAGCACACTGGCGGCCGTTACTAGTCTGAAAACGCTACTTAACCCTTCCGGC
KO_4	CCTCACCAATCCACATTTGCAGTCAACC
KO_5	CCTCCTGAGAGTACACTTGACCA
KO_6	CAGTCCAAACGCAGCGGTATCAC
INF	TGAAGGGCTCCGTTCTCTTTGG
INR	GTGGTAGGATAGGAGACGTAGAATGC
Forward_HindIII	GGATCTAAGCTTCTACGAACTTCCCGCGAGTCAAC
Reverse_BamHI	AGATCCGGATCCGAGCGATTGACCGGTCTCGGGT
Probe_F	ACCCGCCCCCAATACCAGAAAA
Probe_R	TTGAAAGCCGAATTCGAGAC
